# In Situ Casting of Platelet Rich Plasma/SiO2/Alginate for Bone Tissue Engineering Application in Rabbit Mandible Defect Model

**DOI:** 10.30476/DENTJODS.2021.90677.1513

**Published:** 2022-09

**Authors:** Amin Gholijani, Saeid Tavanafar, Nehleh Zareifard, Zahra Vojdani, Mohammad Reza Namavar, Asrin Emami, Tahere Talaei-Khozani

**Affiliations:** 1 Student, Tissue Engineering Lab, Dept. of Anatomy, Shiraz University of Medical Sciences, Shiraz, Iran; 2 Dept. of Oral and Maxillofacial Surgery, School of Dentistry, Shiraz University of Medical Sciences, Shiraz, Iran; 3 Morphometry and Stereology Research Center, Shiraz Medical School, Shiraz University of Medical Sciences, Shiraz, Iran; 4 Dept. of Anatomy, Shiraz University of Medical Sciences, Shiraz, Iran

**Keywords:** Bone, Tissue Engineering, Platelet Rich Plasma, Silicones, Alginate

## Abstract

**Statement of the Problem:**

The administration of both platelet rich plasma (PRP) and silicon dioxide (SiO_2_) to the bone defects accelerates bone repair and regeneration. Appli-cation of both of them may show synergistic regenerative effects

**Purpose:**

Our objective was to evaluate the possible synergistic osteogenic effects of PRP and SiO_2_ by injecting them using an ad hoc device.

**Materials and Method:**

In this experimental study, PRP/SiO_2_ scaffolds were fabricated by in situ casting method with the help of CaCl_2_ as the gelation factor and alginate as the stroma; and then, the biodegradability and spatial arrangement were assessed. The injecta-ble scaffold was introduced into the 40 rabbit mandibular defects by an ad hoc two-channel injecting device. Five defects received PRP/SiO_2_/alginate as the treatment; the other sets of defects were treated by PRP/alginate, SiO_2_/alginate, and the last five defects served as the control groups by getting only alginate injections. The osteogenicity of the scaffolds was evaluated by radiological and histological procedures; they were then compared with each other. Analysis of variance and least significant difference tests were used to analyze the data

**Results:**

The SiO_2_-treated group showed a significant higher bone area compared to PRP/ SiO_2_-treated groups on day 40 (*p*= 0.013). The number of osteocytes was higher in SiO_2_-treated than the control groups on both 20 and 40 days (*p*= 0.032 and 0.022, respectively). The number of osteoclast was also higher in SiO_2_-treated than PRP-treated group (*p*= 0.028). In addition, the cells of this group had just started to create Haversian systems in newly formed bone tissues.

**Conclusion:**

Silica demonstrated a superior osteogenic activity over PRP in both short and long term periods. Evidently, they showed no synergistic regenerative effects. Our ad hoc device was efficiently capable of inserting the scaffolds into the injured sites with no diffi-culties or complications.

## Introduction

The incidence of skeletal defects due to inactivity and obesity, particularly in societies with old population and advanced bone degenerative diseases, has dramatically increased and is expected to double this year [ 1 ]. In addition, the worldwide rate of accidental bone injury had a steeply upward trend over the past few years [ 2 ] and yet it remains a major challenge in the field of orthopedic surgery. Functional defects of the skeletal system usually happen as a result of trauma, injuries and diseases that can cause considerable complications and also various social and economic predicaments [ 3 ]. 

Hence, bone disorders extremely affect the patient's quality of life [ 4 ]. Trauma, cancer, and tuberculosis are the most common problems among the etiologies causing bone defects [ 5 ]. Particularly, mandibular defects are of utmost importance owing to the increasing prevalence and their effect on the matter of facial beauty and elegance. They are usually caused by trauma, removal of mandibular tumors, infection, and congenital diseases [ 6 ]. Surgery and bone grafting are the possible options for treatment of bone defects [ 5 ]. However, autogenous bone grafting is the most common measure to tackle these problems. It is known as the gold standard option due to its remarkable properties such as osteoconduction, osteoinduction, and osteointegrity [ 7 ]. Although autogenous bone grafting is the gold standard modality in treatment of skeletal and specifically mandibular defects, it results in several complications [ 8 - 9 ]. Nowadays, tissue engineering serves as an ideal alternative in dealing with skeletal defects [ 1 ]. Bioactive tissue engineered scaffolds enhance the cell differentiation, proliferation, migration, and angiogenesis, thereby improving ossification and bone formation [ 10 ]. 

One of recently innovative methods in bone tissue engineering is in situ casting of fluid biomaterials in the injured tissue and letting it solidify in the shape of the defect; therefore, we designed an ad hoc device to load biomaterials into the injured site. By using this device, not only is the treating procedure carried out much faster, but also the injected scaffold and the injured site are thoroughly superimposed. Moreover, it is very simple to use this device, and it does not require any specific prefabrication.

Natural biopolymers are widely utilized in this field due to their resemblance with extracellular matrix (ECM), convincing biologic function, and appropriate rate of biodegradability [ 11 ]. Recently, platelet rich plasma (PRP) has been useful in skeletal regenerative medicine due to its effect on accelerating the healing process [ 12 - 14 ]. Evidently, PRP is considered as a rich source of growth factors including platelet-derived growth factor (PDGF); transforming growth factor β (TGF-β); bone morphogenetic proteins (BMPs) as its subset; insulin-like growth factor (IGF-I); and vascular endothelial growth factor (VEGF) which are noticeably effective in angiogenesis, cell differentiation, proliferation and migration [ 14 - 17 ]. Its fibrin fibers form a biodegradable scaffold and are helpful in cell differentiation and proliferation [ 18 ]. Several studies with positive therapeutic results have been conducted using a PRP-based scaffold. For example, using a PRP and hydroxyapatite scaffold led to enhancement of ossification in lumbar vertebrae of rats [ 19 ]. On the other hand, nanoscale bioceramics have proved helpful to enhance cell adhesion, proliferation, and mechanical strength of the scaffold, thereby improving new integrated bone formation [ 11 ]. That is why they are frequently used in bone tissue engineering. They include hydroxyapatite, silicon dioxide (SiO_2_), zirconium dioxide (ZrO_2_), calcium phosphate (Ca_3_(PO_4_)_2_), calcium sulfate (CaSO_4_), and so forth [ 20 - 24 ]. Among these materials, SiO_2_ has drawn the attentions because of enhancing cell adhesions and improving cell viability and proliferation, which are essential for scaffold formation and ossification process [ 25 - 26 ]. According to in vivo studies, SiO_2_, also known as silica, increases proliferation of the endothelial cells and postoperative angiogenesis by accelerating production of VEGF, which plays a significant role in scaffold fabrication and bone formation [ 27 ]. Besides, Silica is involved in accelerating differentiation of the osteoblasts from the osteoprogenitor cells [ 28 - 31 ]. Likewise, subcutaneous transplantation of a combination of nanoporous silica, PRP and type I collagen stimulated the angiogenesis, mineralization and osteogenesis [ 23 ].

Nowadays, researchers prefer to choose the composites of biopolymers and bioceramics as bone tissue engineering material since they present benefits of both groups together in a single scaffold [ 11 ]. Several studies are in favor of their synergistic therapeutic effects [ 20 , 22 , 31 - 32 ]. A combination of collagen, chitosan, and nanoparticles of hydroxyapatite resulted in bone tissue formation with high mechanical strength by increasing the differentiation, proliferation, and adhesion of cells [ 20 ]. A combination of hydroxyapatite and alginate were also used to deliver drugs for boosting osteoblast functions [ 31 ]. In addition, a composite of PRP, hydrox-yapatite and zirconia accelerated the osteogenesis and enhanced number of osteoblasts and osteocytes [ 22 ]. Therefore, we decided to combine PRP, SiO_2_ and alginate and create a composite of biomaterials. The purpose of this study was to evaluate the possible synergic regenerative effects of PRP and silica-alginate injected by ad hoc device in rabbit mandible defect models.

## Materials and Method

### Scaffold fabrication

PRP bank consisted of four 50 mL bags of human platelet serum provided from Fars blood transfusion center (Ghasrodasht Avenue, Shiraz, Fars province, Iran). Heparin was added as an anticoagulant and the number of platelets was estimated to be1.042 ×106/mL. They were aliquoted and frozen for less than 6 months and then thawed to be used in the structure of the scaffold.

PRP was mixed with 1% alginate at the ratio 5:1. Also, 1% SiO_2_ (Silica) nanoparticles (99%, 20-30nm, 25g, US research Nanomaterials, Inc-InterNano. Texas, USA) were prepared and sonicated to disperse the nanoparticles. Then, SiO_2_ was added to the PRP-alginate mixture at a final concentration of 1%. Finally, the liquid was ready to enter one of the cylinders of ad hoc injecting device. On the other hand, 2.5% calcium chloride (Sigma-Aldrich, Inc., USA) was in the other cylinder of the device. Their simultaneous injection into either wells of the culture dish for in vitro examinations or injured site of animal model led to in situ electrogelation and hydrogel formation.

### Scanning electron microscopy (SEM)

Hydrogels formed in culture dishes were prepared for SEM. To do this, the scaffolds were lyophilized (D-375-20, Osterode am Herz, CHRIST, Germany) at -50°C. Gold coating of the samples was done using gold sputter coater (Q150R- ES, Quorum Technologies, UK) and observed by scanning electron microscopy (TESCAN-Vega 3, TESCAN, Czech Republic). Energy dispersive spectroscopy (EDS) was also performed to evaluate the amount of SiO_2_ within the scaffold. The pore size and surface porosity were estimated by image J software (http://imagej.nih.gov/ij/index.html).

### Biodegradability test

Control, PRP, SiO_2_, and PRP/SiO_2_ scaffolds were fabricated in the culture dishes. Calcium chloride 2.5% was added to the scaffolds for electrogelation. Then, they were incubated at 37°C and 5% CO2 for 20 min, so that the hydrogel scaffolds formed firmly. Thereafter, 0.01% trypsin enzyme (Sigma) was added to them, they were incubated for 12 hours, and then their weight was measured. The same procedure was done for the next 24, 48, 72, and 96 hours.

### *In vivo* studies

### Experimental design

There were 4groups in both 20and 40 treatment periods (n=5). The defects in the group 1, also known as the control group, were filled with alginate, while the defects in groups 2, 3 and 4 were loaded with alginate/ SiO_2_, alginate/PRP and alginate/SiO_2_+PRP, respectively. After 20 and 40 days of the follow up, the rabbits were sacrificed and their mandibles were removed.

### Surgery procedure 

20 New Zealand white male rabbits at age three months and weighting about 2 kg were involved in this study. All procedures carried out in this study were in accordance with the approved guidelines of Ethics Committee of Shiraz University of Medical Sciences (IR.SUMS. REC.1397.1074). They were anesthetized by intramuscular injection of Xylazine2% (Alfasan, Woerden-Holland) and ketamine 10% (Bremer PharmaGmbh 34414, Warburg, Germany) with a proportion of 3 to 1, respectively. Next, the mandibular region of samples was shaved and disinfected, using povidone-iodine 10% (DaruDarman Co., Tehran, Iran). Simultaneously, lidocaine HCl 2% (ZEYCO, 401 G1 9020, Mexico) was used to provide local anesthesia and reduce the pain during surgery. The mandibular skin was incised and the masseter muscle was retracted in order to prevent possible injury and expose the mandibular bone. A defect with 9 mm in width, 6 mm in height and 2 mm in depth was created bilaterally in the mandible of rabbits using dental bur and simultaneous irrigation of distilled water. Then, the PRP/alginate/SiO_2_ mixture and Calcium chloride were loaded into each container of the ad hoc device and simultaneously injected into the injured site. Consequently, the defect was filled by in situ casting of the hydrogel scaffold. Finally, the incision was sutured using vicryl 3-0 and nylon 3-0 (Supra Medical Devices Co., Tehran, Iran). The sutured region was sprayed by oxytetracycline (OTC) as postoperative antiseptic.

**Figure 1:**
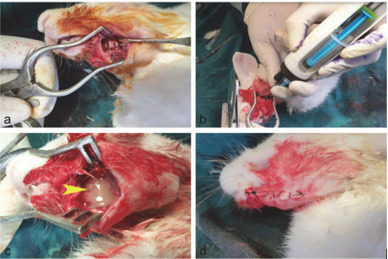
Surgical procedure of the rabbits, **a:** Creation of mandibular bone defect; **b:** Injection of biomaterials into the defect by the ad hoc device; **c:** Gelation of biomaterials (arrowhead); **d:** Suturing of the incision

The operated rabbits became conscious 1 hour after the surgery. They were transported in separate cages for being under control for 20 and 40 days; they had free access to food and water. They received daily intramuscular injection of penicillin/streptomycin for the first three postoperative days (Figure 1).

### Histological assessments

Operated rabbits were sacrificed on day 20 and 40 [ 33 ] according to the defined treatment planning, their mandibles were resected without muscles and fascia. Then, X-ray radiography was done using an X-ray machine (PlanmecaIntra, Finland).

Thereafter, the bones were fixed in buffer formalin 10% in phosphate buffer saline (PBS), and then they were decalcified in HCl 8% and formic acid 8% for 3 days. After, routine tissue processing, 5µm sections were acquired from each block with 300µm interval between each four sections. Therefore, the first four sections belonged to the surface of the defect, whereas the second and third four sections appertained to the middle and end of it, respectively. They were stained by hematoxylin and eosin (H &E). Approximately, 12 images were taken from every H & E stained slide using randomly systematic selected field method. Finally, the total bone and connective tissue areas in addition to the number of osteoblasts, osteocytes, and osteoclasts were estimated by ImageJ software.

### Statistical tests

The data were analyzed using Analysis of variance and least significant difference tests and the p Value less than 0.05 was considered statistically significant. The graphs were depicted and the data analyzed by graph Pad6.

## Results

### *In vitro* evaluations

### SEM

**Figure 2:**
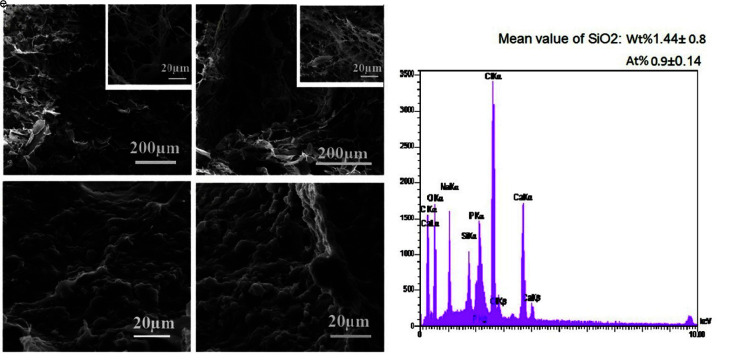
Scanning electron microscopy of the SiO_2_/PRP (A) and PRP (B) scaffolds. Small squares show higher magnification of the scaffold. The MG63 cell line, cultured on both SiO_2_/PRP (C) and PRP (D) scaffolds, showed similar phenotype. EDS confirms the presence of SiO_2_ within the scaffolds (E); SiO_2_, silicon dioxide; PRP, platelet rich plasma; EDS, Energy dispersive spectroscopy

Cell free-scaffolds fabricated with PRP demonstrated a spongy construct with clearly visible fibrin fibers striated in several directions (Figure 2). Cell-free PRP/SiO_2_ scaffolds also indicated a porous structure with more internal density and several accumulations of SiO_2_ nano-particles. The mean value of the pore size was estimated as 334.394±842.687µm2, and the surface porosity was estimated as 45.95%. On the other hand, cell loaded PRP scaffolds showed a similar spongy structure with round cells and few short processes all over the scaffold. In addition, the cells cultured on PRP/SiO_2_ scaffolds had the same appearance. Moreover, EDS test revealed that the percent of weight of SiO_2_ in PRP/SiO_2_ groups was 1.44 ± 0.8.

### Biodegradability test

**Figure 3:**
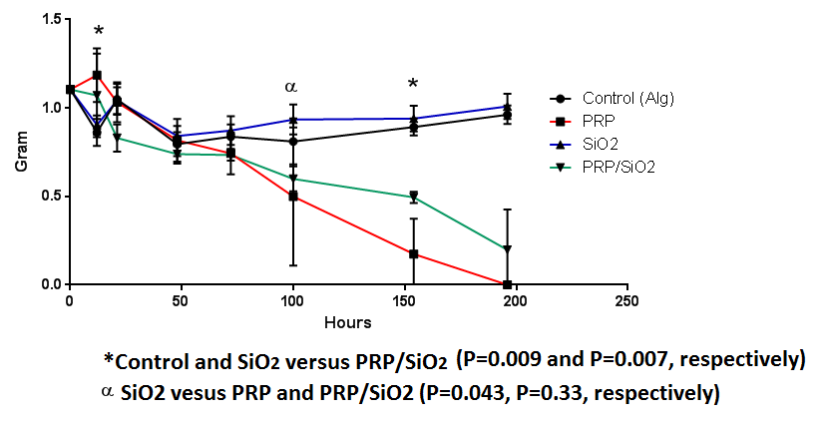
Comparison of the in vitro biodegradation rate of different scaffolds. At the beginning, the presence of PRP decelerated degradation rate, while at the end, PRP-containing scaffolds disintegrated sooner than the control and SiO_2_-containing scaffolds. Alg, alginate; SiO_2_, silicon dioxide; PRP, platelet rich plasma

The data from biodegradation test revealed that at the first hours of incubation, the presence of PRP decelerated biodegradation, while as the time progressed; degradation rate was accelerated by incorporating PRP into the scaffolds (Figure 3).

### *In vivo* studies

#### Gross and radiologicalimages

 On day 20, radiological evaluations showed supreme advantage of PRP/SiO_2_ treated group compared to the other ones. Bone density was the highest in the PRP/SiO_2_ treated group and then it descended to the PRP treated group, SiO_2_ and alginate-treated groups, respectively. Gross images also confirmed the radiological findings. The newly formed bone in the PRP/SiO_2_ treated group was more compact than the other groups were, whereas the other new bony formations were more of a jelly-like structure.

**Figure 4:**
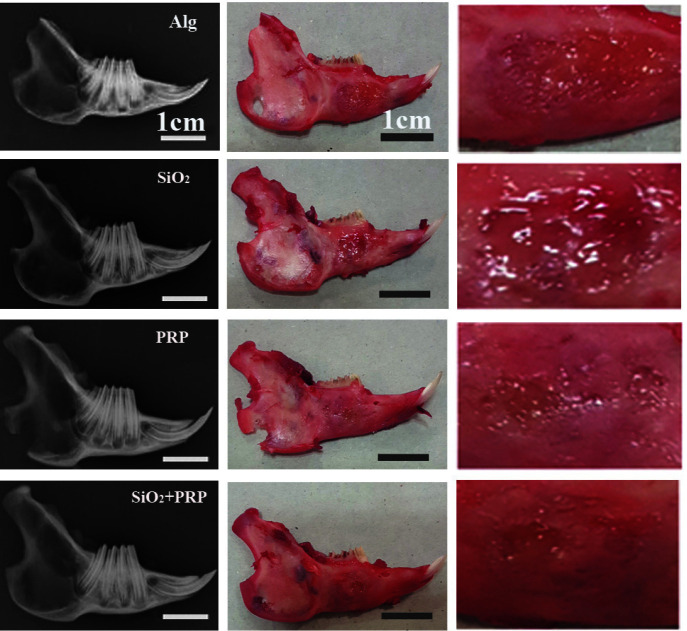
Radiological and gross appearance of the repaired defects in the sample mandibles from various groups 20 days after the surgery. Alg, alginate; SiO_2_, silicon dioxide; PRP, platelet rich plasma

**Figure 5:**
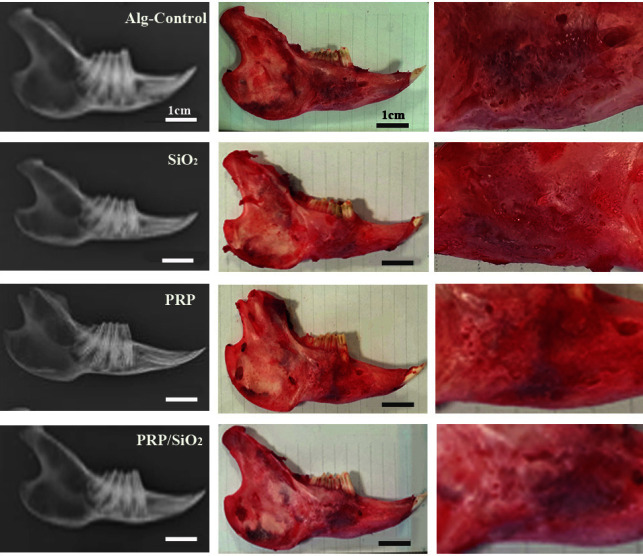
Radiological and gross appearance of the repaired defects in the sample mandibles from different groups 40 days after the surgery. Alg, alginate; SiO_2_, silicon dioxide; PRP, platelet rich plasma

However, the results were somehow different on day 40. Generally, the healing process was better and newly formed bone tissues were more compact and mature compared to day 20. According to the radiology and gross images, the SiO_2_ treated group had the most compact new bone tissue; therefore, it offered the best rehabilitation process. After that, the PRP/SiO_2_, PRP- and alginate-treated groups possessed the better bone density of the new bone tissue, respectively (Figure 4 - Figure 5).

### Histological findings 

**Figure 6:**
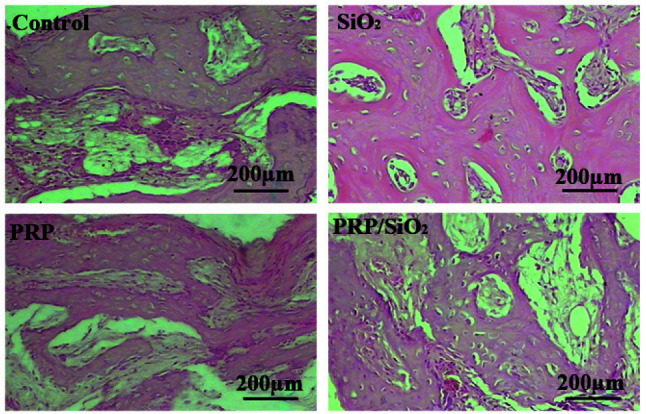
Comparison of the histological sections fromthe mandibular bone defects treated with various scaffolds 20 days after surgery

**Figure 7:**
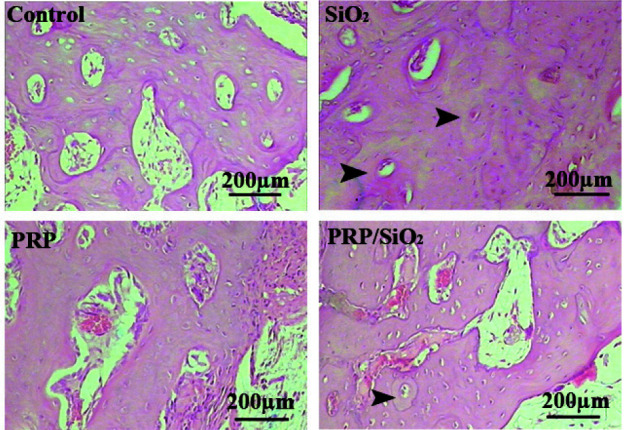
Comparison of the histological sections from the mandibular bone defects treated with various scaffolds 40 days after surgery

**Figure 8:**
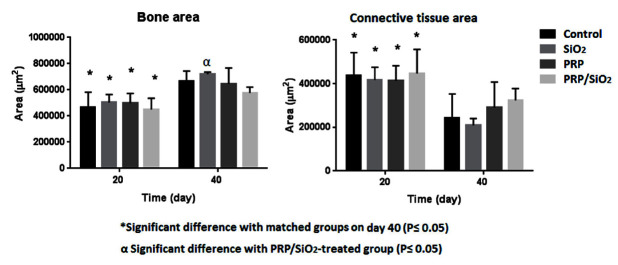
Comparison of bone and connective tissue area in defective part of the mandible from different groups on days 20 and 40 postoperatively

Bone area for each group on day 40 was more than that of the same group on day 20 due to osteogenesis in the 20-day period between days 20 to 40 (Figure 6-Figure 7). There was a significant difference between the matched groups in bone area on days 20 and 40 (*p*= 0.029 for the control versus PRP/SiO_2_ groups, and *p*< 0.01 for the others). On day 20, the SiO_2_ treated group had the most bone area without a significant difference with any other group, while on day 40, the SiO_2_ treated group possessed the most bone area with a significant difference compared to the PRP/SiO_2_ treated group on the same day (*p*=0.013, Figure 8a).

Connective tissue area for each group on day 40 was less than that of the same group on day 20 owing to osteogenesis in the period from days 20 to 40. There was a significant difference between the matched groups in the connective tissue area on days 20 and 40 (*p*= 0.045 for PRP/SiO_2_, *p*= 0.033 for PRP and *p*=0.001 for both control and SiO_2_ versus matched groups). Connective tissue area in the groups treated with PRP/SiO_2_, PRP and alginate was the same statistically on both days 20 and 40 (Figure 8b).

**Figure 9:**
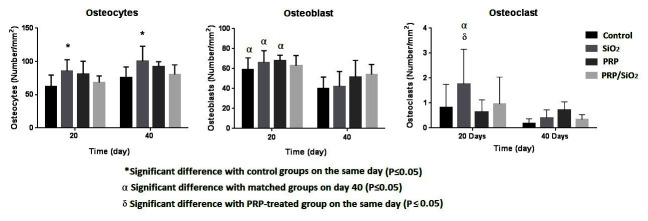
Comparison of the number of osteocytes, osteoblasts, and osteoclasts in defective part of the mandible treated with different scaffolds on days 20 and 40 postoperatively

The number of osteoblasts per µm2 for each group on day 20 was more than that of the same group on day 40 due to either osteoblast migration or differentiation in the 20-day period between days 20 to 40. There was a significant difference between the matched groups in the number of osteoblasts, except for the PRP/SiO_2_ treated group on days 20 and 40 (*p*= 0.022 for the control, *p*= 0.004 for SiO_2_ and *p*= 0.04 for PRP). On day 20, the PRP-treated group had the most osteoblasts without a significant difference with any other group, whereas on day 40, the most osteocytes belonged to the PRP/SiO_2_ treated group without a significant difference with any other group (Figure 9).

The number of osteocytes per µm2 for each group on day 20 was similar to that of the same group on day 40. TheSiO_2_ treated group possessed the most osteocytes on both days 20 (*p*=0.032) and 40 (*p*=0.022) with a significant difference with the control group on the same day (Figure 9).

The number of osteoclasts per µm2 for the control, PRP and PRP/SiO_2_-treated groups was statistically the same on days 20 versus 40. There was a significant difference between the SiO_2_ treated groups on days 20 and 40 (*p*=0.009). In addition, the SiO_2_ treated group had the most osteoclasts on day 20 with a significant difference with the PRP treated group on the same day (*p*=0.028), while on day 40, the most osteoclasts belonged to the PRP treated group without a significant difference with any other group (Figure 9).

## Discussion

In the present study, we designed an injection device to introduce biomaterials for bone repair. This adhocdevice makes the prefabricating scaffolds unnecessary. The applicable value of the injectable biomaterials such as bioactive glass [ 34 ] and hydrogels [ 35 ] has been shown previously. Both PRP and alginate polymerize at the presence of CaCl2. It takes a few minutes to form the PRP gel and we added alginate to accelerate the gelation process in the same form and size of the defect. 

The results of the current study showed that PRP incorporation in the scaffolds led to decelerated degradation rate in short time, whereas, at long term, biodegradation was accelerated. It has been previously reported that platelet-rich fibrin degraded rapidly [ 36 ]. PRP incorporation in the scaffolds led to a decrease in the alginate concentration and it may be responsible for the high rate of degradation. On the other hand, it has been shown that the degradation rate of PRP scaffolds is related to the CaCl2 concentration. As the CaCl2 concentration increases, the degradation rate decreases [ 37 ]. We used 2.5% CaCl2 that led to rapid disintegration of PRP-containing scaffolds. However, in short time, the combination of PRP and alginate decelerated the scaffold disintegration.

In the current study, we observed more bone regeneration and less connective tissue and osteoblasts per µm2 on day 40 compared to day 20. The SiO_2_ treated group had the most regenerated bone area and osteocytes per µm2 on both days 20 and 40. Therefore, SiO_2_ nanoparticles presented themselves as an agent for bone regeneration. Several similar studies have been conducted using the aforementioned agents [ 38 - 40 ]. Biosilica, as a biocompatible, inorganic polymer, has been shown to induce bone formation through enhancing mineralization [ 38 ], angiogenesis [ 39 ] and regulating immunoreactions [ 40 ]. The current work also confirms the positive influence of SiO_2_ on accelerating the bone regeneration.

Combination of the other bioceramic such as HA with organic biomaterials has been shown synergistic impact on bone regeneration [ 19 ]. For instance, a combination of PRP and hydroxyapatite improved the bonenic or inorganic biomaterials to improve the bone repair.

Biphasic mineralized collagen scaffold containing intrafibrillar silica and apatite provoked the mouse mesenchymal stem cells to initiate o steogenesis [ 29 ]. Silicate composite with Graphene/polycaprolactone has been reported to provide a good osteoconductive scaffold for bone regeneration [ 41 ]. SiO_2_/PRP/bone substitute biomaterial has been suggested for replacing the bone, and it was found that SiO_2_ influences in vitro releasing pattern of growth factors by platelet population [ 42 ]. Our previous in vitro study also revealed a composite of SiO_2_ and PRP has led to appropriate osteoblasts viability, proliferation, and function [ 43 ]. In contrast to the previous in vitro investigations, the current study did not indicate synergistic impact on bone regeneration potential by implanting the SiO_2_/PRP/alginate scaffold. Another study was performed utilizing a composite of PRP, mesenchymal stem cells, and nanoporous silicon enclosures as an osteogenic scaffold implanted subcutaneously, which resulted in bone formation and angiogenesis [ 23 ]. However, there are some studies that reject the bone-regenerating characteristics of PRP [ 22 , 44 - 46 ]. For instance, PRP failed to promote bone reconstruction in a canine defect model. In fact, it presented lower amounts of bone formation than the non-PRP group [ 47 ]. In the present study, we observed the same behavior from PRP when it was simultaneously used with silica. Not only it did not enhance the bone reconstruction in the injured site, but it also suppressed the osteogenic activity of silica. Consequently, they do not seem to have synergistic bone regenerative effects.

On the other hand, a PRP and hydroxyapatite/zirconia scaffold accelerated the bone reconstruction and increased the osteoblast and osteocyte counts in the rabbit mandible defect model. Biopsies were harvested at the end of the second, sixth, and eighth postoperative weeks. On the fourteenth postoperative day, the levels of osteogenesis were significantly higher in the hydroxyapatite/ZrO2/PRP-treated group than those of the control groups, whereas at the end of the sixth and eighth postoperative weeks, there were no significant differences between the groups, and the regenerative potential of all the scaffolds was the same [ 22 ]. We chose to evaluate the osteogenic capacity of the scaffolds 20 days after the surgery and we believe that short term assessments may need to find accelerating potential of such combination. 

Our study showed that the combination of SiO_2_ and PRP had no impact on the number of osteoclasts, while in short time, the number of osteoclast increased by SiO_2_ administration. Mesenchymal stem cell differentiation potential into osteoclasts was evaluated on SiO_2_ in combination with CaO scaffolds, and it was found that the differentiation, survival, and adherence of osteoclast precursors were influenced by culturing on the scaffold [ 48 ]. Besides, adding SiO_2_ to poly(lactic-co-glycolic)-acid membrane increases the number of osteoclast in rabbit calvaria defect model [ 49 ]. In another study, the osteoclastogenesis ability of SiO_2_/collagen was compared with hydroxyapatite. Silicone-containing scaffolds increased the bone resorption compared to hydroxy-apatite-containing scaffolds [ 50 ]. On the other hand, PRP has been recorded to inhibit [ 51 - 52 ] or stimulate [ 53 ] the osteoclast differentiation through various mechanisms based on the preparation procedure. In the current study, the number of osteoclasts was similar in the PRP/SiO_2_-, PRP- treated and control animals that may be attributed to the contradictory effects of PRP andSiO_2_ on osteoclastogenesis as well as the way of PRP and SiO_2_ preparation.

The current study had several limitations. Firstly, it was better to sacrifice the rabbits and obtain the samples for radiological and histological assessments in shorter period after the surgery in order to shed light on the probable significant differences in the osteogenic function of the scaffolds. Secondly, radiology images could not be acquired continuously during the postoperative period because of the difficulty in the process of anesthesia and high risk of death of rabbits through it. Thirdly, the calcification rate of the scaffolds could be evaluated to indicate the mineral density and reveal the possible differences between them. Lastly, it would be much more helpful if the gene expressions related to osteogenesis were assessed to clarify the pathways responsible for bone regeneration process.

## Conclusion

That the results of the current study showed that osteogenesis was superior in SiO_2_-treated defects compared to the other groups. The combination of PRP and SiO_2_ did not show any synergistic influence on bone regeneration. Besides, the injectable scaffold could be introduced into the defect by ad hoc device without any adverse impact.

## Acknowledgement

We would like to thank vicechancellor of Shiraz University of Medical Sciences for supporting this work financially (grant no: 10533). The authors wish to thank Dr. Vahedi and the other personnel in the center of com-parative and experimental medicine, Shiraz University of Medical Sciences. We appreciate the collaboration of the personnel in the central lab of Shiraz University and Fars blood transfusion center, Shiraz, Iran. This manuscript was extracted from a thesis by A. Gholijani as a part of fulfillment to obtain MSc degree. The authors would like to thank Shiraz University of Medical Sciences, Shiraz, Iran, and also Center for Development of Clinical Research of Nemazee Hospital and Dr. Nasrin Shokrpour for editorial assistance.

## Conflict of Interest

The authors declare that they have no conflict of interest.
